# Length-Dependent Structural Transformations of Huntingtin PolyQ Domain Upon Binding to 2D-Nanomaterials

**DOI:** 10.3389/fchem.2020.00299

**Published:** 2020-04-21

**Authors:** Mei Feng, David R. Bell, Zhenhua Wang, Wei Zhang

**Affiliations:** ^1^Department of Physics, Institute of Quantitative Biology, Zhejiang University, Hangzhou, China; ^2^Computational Biological Center, IBM Thomas J. Watson Research Center, Yorktown Heights, NY, United States; ^3^School of Materials and Physics, China University of Mining and Technology, Xuzhou, China

**Keywords:** MD simulation, polyQ, graphene, MoS_2_ nanosheet, Huntington's disease (HD)

## Abstract

There is a strong negative correlation between the polyglutamine (polyQ) domain length (Q-length) in the intrinsically disordered Huntingtin protein (Htt) exon-1 and the age of onset of Huntington's disease (HD). PolyQ of Q-length longer than 40 has the propensity of forming very compact aggregate structures, leading to HD at full penetrance. Recent advances in nanobiotechnology provided a new platform for the development of novel diagnosis and therapeutics. Here, we explore the possibility of utilizing 2D-nanomaterials to inhibit the formation of supercompact polyQ structures through the so-called “folding-upon-binding” where the protein structure is dependent on the binding substrate. Using molecular dynamics simulations, we characterize two polyQ peptides with Q-length of 22 (Q22, normal length) and 46 (Q46, typical length causing HD) binding to both graphene and molybdenum disulfide (MoS_2_) nanosheets, which have been applied as antibacterial or anticancer agents. Upon binding, Q22 unfolds and elongates on both grapheme and MoS_2_ surfaces, regardless of its initial conformation, with graphene showing slightly stronger effect. In contrast, initially collapsed Q46 remains mostly collapsed within our simulation time on both nanosheets even though they do provide some “stretching” to Q46 as well. Further analyses indicate that the hydrophobic nature of graphene/MoS_2_ promotes the stretching of polyQ on nanosheets. However, there is strong competition with the intra-polyQ interactions (mainly internal hydrogen bonds) leading to the disparate folding/binding behaviors of Q22 and Q46. Our results present distinct Q-length specific behavior of the polyQ domain upon binding to two types of 2D-nanomaterials which holds clinical relevance for Huntington's disease.

## Introduction

Neurodegenerative Huntington's disease (HD) is caused by expansion of the trinucleotide CAG repeats in exon-1 of the HD gene, the mutation that encodes an extended polyglutamine (polyQ) tract within the N-terminal exon-1 of the Huntingtin protein (Htt) (Macdonald et al., 1993). There is a strong negative correlation between polyQ length (Q-length) and the age of onset of HD (Saudou et al., 1998; Wexler and Res, 2004; Gray et al., 2008). Expanded polyQ regions form oligomers that aggregate into large, insoluble protein complexes, which may subsequently mature to observable fibrils (Perutz et al., 1994). PolyQ length and structure are critical for posited neurotoxicity mechanisms caused by oligomers and larger aggregates (Miller et al., 2011). Recent work from our group has found that at longer Q-lengths, increased β-sheet content seems to promote the formation of supercompact structures (Kang et al., 2017). The increased compactness at long Q-lengths indicates that the polyQ domain may result in increased neural toxicity by inducing distinct morphological changes throughout the entire Htt exon-1 protein (Kang et al., 2017).

It is nontrivial to structurally characterize the Httexon-1 protein due to its inherently disordered nature. It is also known that when the other two domains of Htt exon-1 protein, N17 (the first 17 residues of the Htt N-terminal region) and C38 (38 residues near the Httexon-1 C-terminus, polyproline-rich segment), are absent and the Q-length is 20 or greater, polyQ collapses into a disordered but compact globule (Perutz et al., 1994; Chen et al., 2002; Crick et al., 2006; Miettinen et al., 2012; Heck et al., 2014; Hoop et al., 2016). The supercompact structures of polyQ (not found in other globular proteins) are mainly owing to the “glue-like” propensity of glutamine sidechains to form many hydrogen bonds (Kang et al., 2017). The sidechains tend to be “buried” inside the protein so that the polyQ domains themselves are insoluble.

Meanwhile, there is a growing interest in applying nanobiotechnology for biomedical applications. For example, biocompatible two-dimensional (2D) nanomaterials with unique compositional, structural and physicochemical features, such as graphene and MoS_2_, have gained increased attention in the biomedical field as potential antibacterial and antitumor agents. In this study, we explore the possibility of interrupting the formation of a supercompact polyQ using novel engineered nanomaterials like graphene or MoS_2_. Some intrinsically disordered proteins (Arai et al., 2015; Shammas et al., 2016; Bonetti et al., 2018) and switch sequences (Chen and Elber, 2014; Chen et al., 2016; Porter and Looger, 2018) are thought to utilize the folding-upon-binding mechanism where the protein structure is dependent on the binding substrate. Both graphene and MoS_2_ nanosheets have a highly hydrophobic surface with large water contact angles (despite that MoS_2_ has partial charges on Mo and S atoms), offering a biomimetic environment for protein binding when interfaced with water (Mathesh et al., 2016; Gu et al., 2017; Zhang et al., 2017). Previous studies have observed a variety of effects of graphene on proteins, including disruption of protein structures (Zuo et al., 2011; Chong et al., 2015; Wang et al., 2015), enhancement of enzymatic activity (Mathesh et al., 2016), and potential interference of protein-protein interactions (Luan et al., 2015, 2016a; Feng et al., 2016). Comparatively, there are less studies on MoS_2_'s effect on proteins partly due to the lack of appropriate potential function parameters for MoS_2_ simulation. The recently developed MoS_2_ force field parameters that are compatible with the TIP3P water model (Luan and Zhou, 2016) might help facilitate a wider application of computer simulations on the interaction of MoS_2_ with biomolecules.

Herein, we use all-atom molecular dynamics (MD) simulations to study Htt-polyQ adsorption onto graphene and MoS_2_ nanosheet. We investigate two poly glutamine-lengths, 22 (Q22, healthy Q-length) and 46 (Q46, typical Q-length associated with HD at full penetrance) to determine if the binding mechanism of the polyQ on these nanosheets is Q-length dependent. We find that Q22 exhibits a similar binding mode on both graphene and MoS_2_ surfaces (with graphene showing a slightly stronger effect) regardless of its initial configuration - the final conformation of Q22 is fully extended. On the other hand, the initially collapsed Q46 remains mostly collapsed when adsorbing onto graphene or MoS_2_ nanosheet, indicating that neither graphene nor MoS_2_ is sufficient to fully stretch the supercompact Q46 structure (again with graphene showing slightly stronger effect). Further energetic and hydrogen bonding analyses indicate that the difference in Q22 vs. Q46 binding conformations is determined by the competition between polyQ internal interactions (mainly intra-hydrogen-bonds) and polyQ-nanosheet interactions. Insights derived from our simulations of polyQ interaction with these novel 2D-nanomaterials may lead to future potential applications in diagnoses and therapeutics for Huntington's disease (HD).

## Simulation Methods

**Figure 1:**
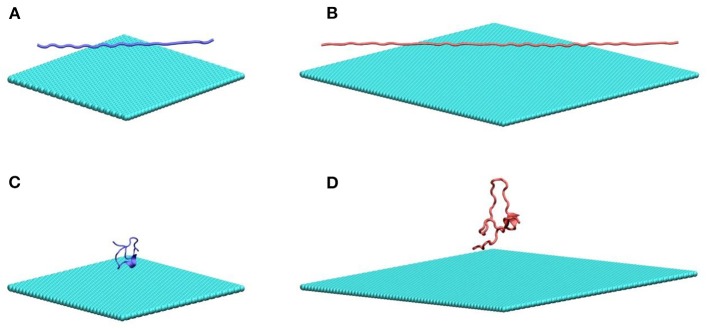
MD simulation systems of a fully extended **(A)** and collapsed **(B)** Q22 peptide near the water–graphene interface, and a fully extended **(C)** and collapsed **(D)** Q46 near the water–graphene interface. Atoms in graphene and PolyQ are shown as spheres and cartoon, respectively. Water molecules and ions are not shown. Graphene is colored in cyan; the Q22 is colored in blue; and the Q46 is colored in red. The equivalent simulation systems of polyQ on the MoS_2_ surface are shown in Figure S1.

In this work, we study two poly glutamine peptides of length 22 (Q22) and 46 (Q46) adsorbing onto graphene and MoS_2_ nanosheets. Figure 1 illustrates the initial configurations of polyQ on the graphene surface simulation systems: fully extendedQ22 at the graphene interface (Figure 1A), collapsed Q22 at the graphene interface (Figure 1B), fully extended Q46 at the graphene interface (Figure 1C), and collapsed Q46 at the graphene interface (Figure 1D). The equivalent simulation systems of polyQ on the MoS_2_ surface are shown in Figure S1. In each initial configuration, the polyQ was separated from the graphene/MoS_2_ nanosheet by 0.75 nm to prevent large initial interactions. The OPLS-AA force field (MacKerell et al., 1998) was used for the polyQ. The force fields for graphene and MoS_2_ nanosheets are the same as in our previous MD studies (Tu et al., 2013; Luan and Zhou, 2016; Luan et al., 2016b). Sodium and chlorine ions were added in each system to yield an electrolyte concentration of 100 mM. The TIP3P model (Jorgensen et al., 1983; Neria et al., 1996) was used for water and the standard force field was used for ions (Beglov and Roux, 1994).

All MD simulations were performed with GROMACS (Hess et al., 2008). To investigate both adsorption of Q22 and Q46 on the nanosheets as well as to capture protein conformational changes, each simulation was run for 600 ns for Q22 and 1000 ns for Q46 under the NVT (T = 300 K) ensemble. Following similar protocols used in our previous studies (Zhou et al., 2001, 2004; Zhou, 2003, 2004; Li et al., 2005; Das et al., 2009; Xia et al., 2012), we first performed energy minimization and 100 ps MD equilibration under the NPT ensemble (pressure = 1 bar) prior to production runs with the positions of protein and nanosheets restrained to relax the solvent. A smooth cutoff (cutoff distance of 12 Å) was used to calculate the van der Waals (vdW) interactions. The particle-mesh Ewald method (with the grid size ~1 Å) was applied for the electrostatic interactions. The motion equation was integrated using the Verlet algorithm, with a time step of 2 fs. The v-rescale temperature coupling method was employed in our simulations (Bussi et al., 2007).

## Results

**Figure 2:**
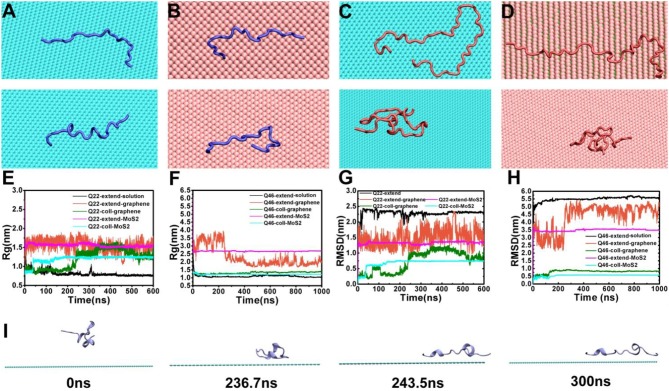
**(A,B)** The final conformations of the initially fully extended (top) and collapsed (bottom) Q22 on graphene **(A)** and MoS_2_ **(B)** nanosheets. **(C,D)** the final conformations of initially fully extended (top) and collapsed (bottom) Q46 on graphene **(C)** and MoS_2_ **(D)** nanosheets. **(E,F)** The radius of gyration (Rg) of Q22 **(E)** and Q46 **(F)**. **(G,H)** The root-mean-square deviation of atomic positions (RMSD) of Q22 **(G)** and Q46 **(H)**. Data were obtained from the following trajectories: initially fully extended Q22/Q46 alone in water (black), initially fully extended Q22/Q46 on graphene (red), initially collapsed Q22/Q46 on graphene (green), initially fully extended Q22/Q46 on MoS_2_ (purple), and initially collapsed Q22 on MoS_2_ (blue). **(I)** Snapshots of initially collapsed Q22 binding and extension onto graphene from the simulation trajectory.

To investigate the influence of graphene and MoS_2_ nanosheets on the polyQ structure, we calculated the radius of gyration (Rg) and root-mean-square deviation (RMSD) of Q22 and Q46 as a function of time (Figure 2). For comparison, we also analyzed the trajectories of the control systems containing either Q22 or Q46 alone in aqueous solution. For the control systems, the polyQ peptide starts from an initially fully extended configuration with the Rg value of 2.76 nm for Q22 and 5.91 nm for Q46. As the polyQ peptides collapse in solution, the Rg decreases to stabilize at 0.76 nm for Q22 and 1.05 nm for Q46 (black in Figures 2E,F). In the polyQ + graphene and polyQ + MoS_2_ systems, the initially fully extended Q22 remains extended with the Rg fluctuates around 1.52 nm (red and purple in Figure 2E). However, the initially collapsed Q22 unfolds and extends across the nanosheet surfaces, as also evidenced by the jump in Rg from ~0.90 to 1.25 nm, with graphene showing slightly larger Rg than MoS_2_ (green and blue in Figure 2E). In contrast, the collapsed Q46 peptide does not unfold when it binds to the nanosheets. Rather, the Rg of the initially collapsed Q46 increases somewhat and fluctuates around 1.36 nm when bound to graphene and around 1.22 nm when bound to MoS_2_ (the Rg of the equivalent control system is 1.05 nm), again indicating graphene has slightly stronger denaturing capability toward Q46 than MoS_2_. The Rg of the initially fully extended Q46 is 1.94 nm on graphene and 2.67 nm on MoS_2_. The smaller Rg of the initially fully extended Q46 on graphene is a result of the extended peptide “winding back” toward itself, which should not be over-read in terms of significance, but rather as an indication of non-convergence in our simulations for the fully extended case. The collapsed case, on the other hand, should be more indicative. Collectively, these results indicate that the 2D-nanosheets can induce unfolding of the Q22 structure, but not Q46, with graphene showing slightly stronger capacity, which is consistent with previous studies where graphene displays stronger interaction with biomolecules than MoS_2_ (Tu et al., 2013; Luan and Zhou, 2018; Wu et al., 2018).

The RMSD “trajectories” largely mimic the Rg results, namely there are RMSD jumps for the Q22 collapsed systems indicating unfolding (again with graphene showing somewhat larger jumps), while the rest of the systems fluctuates mostly randomly. The exception is when the initially fully extended Q22 and Q46 systems bind to graphene, their RMSDs fluctuate more widely when compared to the control systems, indicating large configurational diversity.

Figures 2A–D show the representative final configurations of Q22 and Q46 on the graphene and MoS_2_ surfaces. Both the initially fully stretched and collapsed Q22 adsorbed onto the hydrophobic graphene and MoS_2_ nanosheets in a similar stretched manner, while the initially collapsed Q46 did not stretch out on either of the nanosheets like the collapsed Q22 did. For the graphene system, the collapsed Q22 starts to unfold and spread out on the surface of the graphene after 236 ns (Figure 2I). This transition process corresponds to the increase of Rg shown in Figure 2E at ~236 ns. At 244 ns, the Rg reaches 1.27 nm, which is correlated with Q22 achieving a stretched configuration on graphene. Note that this configuration is not fully stretched but maintains some kinks and turns and remains stable for over 50 ns. The collapsed Q22 on MoS_2_ nanosheet exhibits similar behavior to that of graphene except the unfolding is not as extended. However, the collapsed Q46 does not unfold on either the graphene or MoS_2_ nanosheet (Figures 2C,D, bottom panel).

In order to further characterize the structure of polyQ on the surface of nanosheets, we analyzed the contacts between the polyQ peptides and the nanosheets. This involved: (1) calculating the number of heavy atoms in contact with graphene and MoS_2_ nanosheets (Figures S2A,B), (2) computing the average center of mass (COM) distances between polyQ sidechains and graphene, and between polyQ sidechains and the upper sulfur atoms in the MoS_2_ sheet (Figures S2C,D), as a function of simulation time. Here, a contact between polyQ and nanosheets was defined if the distance between any polyQ heavy atoms is within a distance of 4.5 Å of any graphene/MoS_2_ atoms. In both the graphene and MoS_2_ systems, the atom contact number (ACN) and the COM distance of the initially fully extended Q22 show that Q22 quickly binds the nanosheets (<20 ns). The ACN and COM distance of the initially collapsed Q22 reveal more gradual, detailed binding and unfolding behavior. The initially collapsed Q22 systems exhibit two distinct binding stages: (i) In the early binding stage (0–236 ns in graphene, and 0–40 ns in MoS_2_), a portion of the Q22 atoms are in direct contact with the graphene and MoS_2_ nanosheets. The binding is not strong enough to restrain the Q22 motion, so the Q22 still diffuses along the graphene/MoS_2_ surface, while the number of contacting atoms slowly increases. (ii) This is the stable binding stage (after 244 ns in graphene, and after 136 ns in MoS_2_) where the ACN reaches the maximum, which is very close to the stable binding stage of the fully extended Q22. For the Q46 system, the final ACN of the initially collapsed Q46 are far less than the initially fully extended Q46, and the COM distances with the graphene (0.67 nm) and MoS_2_ (0.92 nm) surface are far away from the initially fully extended Q46 (0.42 nm on graphene and 0.33 nm on MoS_2_), indicating different nanosheet binding structures of Q46 based on different initial conformations or non-convergence in simulations (unfortunately, a fully convergent simulation is beyond our current reach with limited computational resources. Nevertheless, we believe the Q-length dependent behavior is clear even with the current simulation lengths).

**Figure 3:**
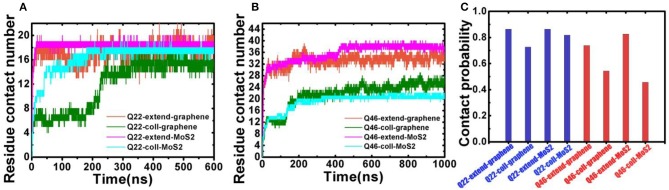
**(A)** Residue contact number between Q22 and graphene nanosheet, and between Q22 and the upper S layer in the MoS_2_ sheet. **(B)** Residue contact number between Q46 and graphene nanosheet, and between Q46 and the upper S layer in the MoS_2_ sheet. **(C)** The contact ratio of Q22 (blue) and Q46 (red) with graphene and MoS_2_ nanosheets.

The binding behavior of polyQ on 2D-nanosheetsis further characterized by monitoring the residue contacts and contact ratio of polyQ with nanosheets (Figure 3). The contact ratio is defined as the number of residues in contact with the nanosheet to the total number of residues. A residue is in contact with the nanosheet if any heavy atom is within 4.5 Å of the nanosheet. In the graphene system, the average residue contact number (RCN) of the initially collapsed Q22 in contact with the nanosheetis ~16 with a contact ratio of 73%, close to the initially fully extended Q22 system with ~19 contacting residues and 86% contact ratio (average of the last 300 ns of trajectory). The RCN of the initially collapsed Q46 on graphene is ~25 with a contact ratio of 54%, far less than the initially fully extended Q46 system with ~34 contacting residues and 74% contact ratio. In the MoS_2_ systems, the RCN of the initially collapsed Q22 in contact with the nanosheet is ~18 and the contact ratio is 82%, similar to the initial fully extended Q22 system. The RCN and contact ratio of the initially collapsed Q46 on MoS_2_ is ~21 and 46%, far less than the initially fully extended Q46 values of ~38 and 83%. The residue contact and contact ratio analyses indicate that when polyQ binds to the graphene/MoS_2_ surface, most of the Q22 residues are in contact with the 2D-nanomaterials, typical of an unfolded, extended structure. In contrast, only part of the collapsed Q46 is in contact with the 2D-nanomaterials, so although the nanosheets induce an unfolding of Q22, they do not cause a full unfolding of Q46 within our simulation time.

**Figure 4:**
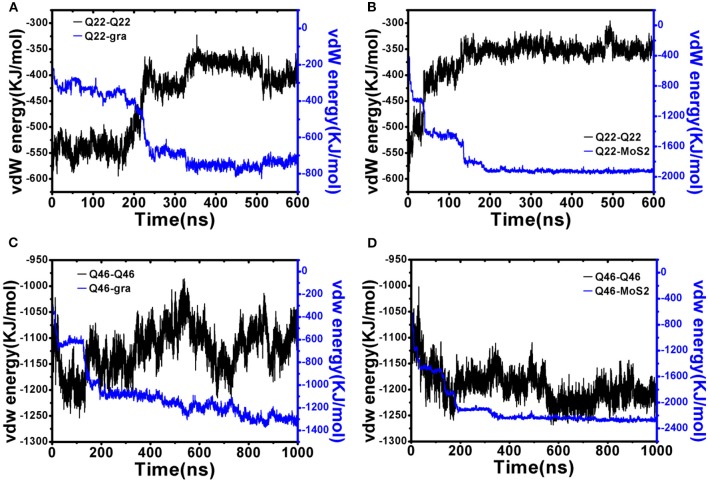
Interaction energy analyses of initially collapsed Q22 and Q46 on graphene and MoS_2_ nanosheet. **(A,B)** Q22 self vdW interaction energy compared to Q22-nanosheet vdW interaction energy for graphene **(A)** and MoS_2_ **(B)**. **(C,D)** Q46 self vdW interaction energy compared to Q46-nanosheet vdW interaction energy for graphene **(C)** and MoS_2_ **(D)**.

Next, we performed energetic analyses to gain a deeper understanding on the interactions between polyQ and nanosheets as well as among polyQ atoms (self-interaction). Although glutamine is normally a hydrophilic residue, isolated polyQ in water shows strong hydrophobic protein properties, including the propensity of forming a supercompact structure and self-aggregation. Therefore, polyQ adsorption onto the graphene/MoS_2_ nanosheet should be favorable due to hydrophobic forces. This favorable binding is also clearly indicated in the time-dependent van der Waals (vdW) interaction energies between polyQ and graphene/MoS_2_ nanosheets (Figure 4; here we used vdW energy for comparison as graphene has no partial charges on C atoms and the electrostatic energy is actually very small compared to vdW for MoS_2_ due to its symmetry even though both Mo and S have partial charges; The electrostatic interaction energy between Qs is shown in Figure S3). For example, as shown in Figure 4A, when the collapsed Q22 unfolds and extends on the graphene nanosheet, there is a significant loss of the self vdW energy and a larger gain in the Q22-graphene interaction energy. Same for the Q22 on MoS_2_ nanosheet (Figure 4B). On the other hand, for the collapsed Q46, the self vdW energy remains strong without any significant loss, indicating no meaningful unfolding on either graphene or MoS_2_. Again, these results indicate that the hydrophobic nature of graphene and MoS_2_ is energetically favorable for the unfolding of Q22 on the nanosheet surface, but probably not strong enough to unfold Q46.

PolyQ peptides are known to form hydrogen bonds using both the glutamine backbone and sidechain which is the main cause of the formation of polyQ supercompact structures (Perutz et al., 1994; Chen et al., 2002; Crick et al., 2006; Miettinen et al., 2012; Heck et al., 2014; Hoop et al., 2016; Kang et al., 2017). Here, we analyze the time evolution of polyQ hydrogen bonds and find interesting competition behavior between the intra-polyQ interactions (mainly hydrogen bonds) and polyQ sidechain-nanosheet interactions. Figures S4, S5 show the number of all hydrogen bonds (A and B) and sidechain-sidechain hydrogen bonds (C and D) of Q22 and Q46 for the graphene and MoS_2_ simulations. The polyQ sidechain-nanosheet interaction is sufficiently strong in the Q22 systems to not only prevent the collapse of Q22 but also to unfold collapsed Q22, reducing the number of intra-chain hydrogen bonds. However, the polyQ sidechain-nanosheet interaction is not strong enough to unfold the collapsed Q46 though it can prevent the collapse of initially extended Q46 on graphene and MoS_2_. Therefore, from our work, we find that the two-dimensional nanomaterial graphene and MoS_2_ have the potential to inhibit the collapse of polyQ, but not for Q-lengths up to Q46.

## Conclusion

To summarize, we have investigated polyQ peptides of two lengths (22 and 46) binding to both graphene and MoS_2_ nanosheets using atomistic molecular dynamics simulations. Two different initial polyQ configurations, fully extended and collapsed, were employed to identify structure dependent folding/binding behavior. Our simulations reveal that both Q22 and Q46 bind to graphene and MoS_2_ nanosheets effectively, but in a Q-length dependent manner. Upon binding, Q22 unfolds and elongates onto both 2D-nanomaterial surfaces, regardless of the initial conformation, with graphene displaying slightly stronger effect. In contrast, Q46 does not spontaneously stretch out onto the graphene/MoS_2_ surfaces within our simulation time if starting from an initially collapsed structure. Detailed analyses indicate that the differential binding behavior of Q22 and Q46 is due to competition between the hydrophobic polyQ-nanosheet interactions and internal polyQ-polyQ self-interactions (mostly intra-hydrogen-bonds). We believe our current work reveals important polyQ length-dependent folding/binding behavior upon binding to novel 2D-nanomaterials, which might have implications for future potential clinical applications to Huntington's disease.

## Data Availability Statement

All datasets generated for this study are included in the article/Supplementary Material.

## Author Contributions

MF: job design, MD simulation, data collection, draft paper, and approve final paper for publication. DB and WZ: make important changes to the paper and approve final paper for publication. ZW: data collection and approve final paper for publication.

## Conflict of Interest

DB was employed by the company IBM Thomas J. Watson Research Center. The remaining authors declare that the research was conducted in the absence of any commercial or financial relationships that could be construed as a potential conflict of interest. The handling Editor declared a shared affiliation, though no other collaboration, with one of the authors DB.

## References

[B1] Arai M. Sugase K. Dyson H. J. Wright P. E. (2015). Conformational propensities of intrinsically disordered proteins influence the mechanism of binding and folding. Proc. Natl. Acad. Sci. U. S. A. 112, 9614–9619. 10.1073/pnas.1512799112 26195786 PMC4534220

[B2] Beglov D. Roux B. (1994). Finite representation of an infinite bulk system - solvent boundary potential for computer-simulations. J. Chem. Phys. 100, 9050–9063. 10.1063/1.466711

[B3] Bonetti D. Troilo F. Brunori M. Longhi S. Gianni S. (2018). How robust is the mechanism of folding-upon-binding for an intrinsically disordered protein? Biophys. J. 114, 1889–1894. 10.1016/j.bpj.2018.03.017 29694866 PMC5937165

[B4] Bussi G. Donadio D. Parrinello M. (2007). Canonical sampling through velocity rescaling. J. Chem. Phys. 126:014101. 10.1063/1.2408420 17212484

[B5] Chen S.-H. Elber R. (2014). The energy landscape of a protein switch. Phys. Chem. Chem. Phys. 16, 6407–6421. 10.1039/c3cp55209h 24473276

[B6] Chen S.-H. Meller J. Elber R. (2016). Comprehensive analysis of sequences of a protein switch. Protein Sci. (2016) 25, 135–146. 10.1002/pro.2723 26073558 PMC4815306

[B7] Chen S. M. Ferrone F. A. Wetzel R. (2002). Huntington's disease age-of-onset linked to polyglutamine aggregation nucleation. Proc. Natl. Acad. Sci. U. S. A 99, 11884–11889. 10.1073/pnas.182276099 12186976 PMC129363

[B8] Chong Y. Ge C. Yang Z. Garate J. A. Gu Z. Weber J. K.. (2015). Reduced cytotoxicity of graphene nanosheets mediated by blood-protein coating. ACS Nano (2015) 9, 5713–5724. 10.1021/nn5066606 26040772

[B9] Crick S. L. Jayaraman M. Frieden C. Wetzel R. Pappu R. V. (2006). Fluorescence correlation spectroscopy shows that monomeric polyglutamine molecules form collapsed structures in aqueous solutions. Proc. Natl. Acad. Sci. U. S. A. 103, 16764–16769. 10.1073/pnas.0608175103 17075061 PMC1629004

[B10] Das P. Li J. Royyuru A. K. Zhou R. (2009). Free energy simulations reveal a double mutant avian H5n1 virus hemagglutinin with altered receptor binding specificity. J. Comp. Chem. 30, 1654–1663. 10.1002/jcc.21274 19399777

[B11] Feng M. Kang H. Yang Z. Luan B. Zhou R. (2016). Potential disruption of protein-protein interactions by graphene oxide. J. Chem. Phys. 144:225102. 10.1063/1.4953562 27306022

[B12] Gray M. Shirasaki D. I. Cepeda C. Andre V. M. Wilburn B. Lu X.-H.. (2008). Full-length human mutant huntingtin with a stable polyglutamine repeat can elicit progressive and selective neuropathogenesis in bachd mice. J. Neurosci. (2008) 28, 6182–6195. 10.1523/JNEUROSCI.0857-08.2008 18550760 PMC2630800

[B13] Gu Z. De Luna P. Yang Z. Zhou R. (2017). Structural influence of proteins upon adsorption to Mos_2_ nanomaterials: comparison of Mos_2_ force field parameters. Phys. Chem. Chem. Phys. 19, 3039–3045. 10.1039/C6CP05260F 28079199

[B14] Heck B. S. Doll F. Hauser K. (2014). Length-dependent conformational transitions of polyglutamine repeats as molecular origin of fibril initiation. Biophys. Chem. (2014) 185, 47–57. 10.1016/j.bpc.2013.11.008 24333917

[B15] Hess B. Kutzner C. van der Spoel D. Lindahl E. (2008). Gromacs 4: algorithms for highly efficient, load-balanced, and scalable molecular simulation. J. Chem. Theory Comp. 4, 435–447. 10.1021/ct700301q 26620784

[B16] Hoop C. L. Lin H.-K. Kar K. Magyarfalvi G. Lamley J. M. Boatz J. C.. (2016). Huntingtin exon 1 fibrils feature an interdigitated beta-hairpin-based polyglutamine core. Proc. Natl. Acad. Sci. U. S. A. 113, 1546–1551. 10.1073/pnas.1521933113 26831073 PMC4760812

[B17] Jorgensen W. L. Chandrasekhar J. Madura J. D. Impey R. W. Klein M. L. (1983). Comparison of simple potential functions for simulating liquid water. J. Chem. Phys. 79, 926–935. 10.1063/1.445869

[B18] Kang H. Vazquez F. X. Zhang L. Das P. Toledo-Sherman L. Luan B.. (2017). Emerging beta-sheet rich conformations in supercompact huntingtin exon-1 mutant structures. J. Am. Chem. Soc. 139, 8820–8827. 10.1021/jacs.7b00838 28609090 PMC5835228

[B19] Li J. Liu T. Li X. Ye L. Chen H. Fang H.. (2005). Hydration and dewetting near graphite-Ch(3) and graphite-cooh plates. J. Phys. Chem. B 109, 13639–48. 10.1021/jp044090w 16852709

[B20] Luan B. Huynh T. Zhou R. (2016a). Potential interference of protein-protein interactions by graphyne. J. Phys. Chem. B 120, 2124–2131. 10.1021/acs.jpcb.5b11449 26885561

[B21] Luan B. Tien H. Zhao L. Zhou R. (2015). Potential toxicity of graphene to cell functions via disrupting protein-protein interactions. ACS Nano 9, 663–669. 10.1021/nn506011j 25494677

[B22] Luan B. Tien H. Zhou R. (2016b). Complete wetting of graphene by biological lipids. Nanoscale 8, 5750–5754. 10.1039/C6NR00202A 26910517

[B23] Luan B. Zhou R. (2016). Wettability and friction of water on a Mos_2_ nanosheet. Appl. Phys. Lett. 108:131601 10.1063/1.4944840

[B24] Luan B. Zhou R. (2018). Spontaneous transport of single-stranded DNA through graphene-Mos_2_ heterostructure nanopores. ACS Nano 12, 3886–3891. 10.1021/acsnano.8b01297 29648440

[B25] Macdonald M. E. Ambrose C. M. Duyao M. P. Myers R. H. Lin C. Srinidhi L. (1993). A novel gene containing a trinucleotide repeat that is expanded and unstable on huntingtons-disease chromosomes. Cell 72, 971–983. 10.1016/0092-8674(93)90585-E 8458085

[B26] MacKerell A. D. Bashford D. Bellott M. Dunbrack R. L. Evanseck J. D. Field M. J.. (1998). All-atom empirical potential for molecular modeling and dynamics studies of proteins. J. Phys. Chem. B 102, 3586–3616. 10.1021/jp973084f 24889800

[B27] Mathesh M. Luan B. Akanbi T. O. Weber J. K. Liu J. Barrow C. J. (2016). Opening lids: modulation of lipase immobilization by graphene oxides. ACS Catalysis 6, 4760–4768. 10.1021/acscatal.6b00942

[B28] Miettinen M. S. Knecht V. Monticelli L. Ignatova Z. (2012). Assessing polyglutamine conformation in the nucleating event by molecular dynamics simulations. J. Phys. Chem. B 116, 10259–10265. 10.1021/jp305065c 22770401

[B29] Miller J. Arrasate M. Brooks E. Libeu C. P. Legleiter J. Hatters D.. (2011). Identifying polyglutamine protein species *in situ* that best predict neurodegeneration. Nat. Chem. Biol. 7:925. 10.1038/nchembio.694 22037470 PMC3271120

[B30] Neria E. Fischer S. Karplus M. (1996). Simulation of activation free energies in molecular systems. J. Chem. Phys. 105, 1902–1921. 10.1063/1.472061

[B31] Perutz M. F. Johnson T. Suzuki M. Finch J. T. (1994). Glutamine repeats as polar zippers - their possible role in inherited neurodegenerative diseases. Proc. Natl. Acad. Sci. U. S. A. 91, 5355–5358. 10.1073/pnas.91.12.5355 8202492 PMC43993

[B32] Porter L. L. Looger L. L. (2018). Extant fold-switching proteins are widespread. Proc. Natl. Acad. Sci. U. S. A. 115, 5968–5973. 10.1073/pnas.1800168115 29784778 PMC6003340

[B33] Saudou F. Finkbeiner S. Devys D. Greenberg M. E. (1998). Huntingtin acts in the nucleus to induce apoptosis but death does not correlate with the formation of intranuclear inclusions. Cell 95, 55–66. 10.1016/S0092-8674(00)81782-1 9778247

[B34] Shammas S. L. Crabtree M. D. Dahal L. Wicky B. I. M. Clarke J. (2016). Insights into coupled folding and binding mechanisms from kinetic studies. J. Biol. Chem. 291, 6689–6695. 10.1074/jbc.R115.692715 26851275 PMC4807256

[B35] Tu Y. Lv M. Xiu P. Huynh T. Zhang M. Castelli M.. (2013). Destructive extraction of phospholipids from *Escherichia Coli* membranes by graphene nanosheets. Nat. Nanotechnol. 8, 594–601. 10.1038/nnano.2013.125 23832191

[B36] Wang X. Weber J. K. Liu L. Dong M. Zhou R. Li J. (2015). A novel form of beta-strand assembly observed in a beta(33-42) adsorbed onto graphene. Nanoscale 7, 15341–15348. 10.1039/C5NR00555H 26331805

[B37] Wexler N. S. Res U. S. V. C. (2004). Venezuelan kindreds reveal that genetic and environmental factors modulate Huntington's disease age of onset. Proc. Natl. Acad. Sci. U. S. A. 101, 3498–3503. 10.1073/pnas.0308679101 14993615 PMC373491

[B38] Wu R. Ou X. Tian R. Zhang J. Jin H. Dong M.. (2018). Membrane destruction and phospholipid extraction by using two-dimensional Mos_2_ nanosheets. Nanoscale 10, 20162–20170. 10.1039/C8NR04207A 30259040

[B39] Xia Z. Clark P. Huynh T. Loher P. Zhao Y. Chen H.-W.. (2012). Molecular dynamics simulations of ago silencing complexes reveal a large repertoire of admissible ‘seed-less' targets. Sci. Rep. 2:569. 10.1038/srep00569 22888400 PMC3415692

[B40] Zhang L. Feng M. Zhou R. Luan B. (2017). Structural perturbations on Huntingtin N17 domain during its folding on 2d-nanomaterials. Nanotechnology 28:354001. 10.1088/1361-6528/aa7ba5 28649967

[B41] Zhou R. (2003). Trp-Cage: folding free energy landscape in explicit water. Proc. Natl. Acad. Sci. U. S. A. 100, 13280–5. 10.1073/pnas.2233312100 14581616 PMC263783

[B42] Zhou R. (2004). Exploring the protein folding free energy landscape: coupling replica exchange method with P3me/Respa algorithm. J. Mol. Graph. Model. 22, 451–63. 10.1016/j.jmgm.2003.12.011 15099840

[B43] Zhou R. Berne B. J. Germain R. (2001). The free energy landscape for B hairpin folding in explicit water. Proc. Natl. Acad. Sci. 98, 14931–14936. 10.1073/pnas.201543998 11752441 PMC64961

[B44] Zhou R. Huang X. Margulis C. J. Berne B. J. (2004). Hydrophobic collapse in multidomain protein folding. Science (2004) 305, 1605–9. 10.1126/science.1101176 15361621

[B45] Zuo G. Zhou X. Huang Q. Fang H. Zhou R. (2011). Adsorption of villin headpiece onto graphene, carbon nanotube, and C60: effect of contacting surface curvatures on binding affinity. J. Phys. Chem. 115, 23323–23328. 10.1021/jp208967t

