# Supplementary material for: Length-Dependent Structural Transformations of Huntingtin PolyQ Domain Upon Binding to 2D-Nanomaterials
Source: Front Chem. 2020 Apr 21;8:299. doi: 10.3389/fchem.2020.00299 (PMC7189795; doi:10.3389/fchem.2020.00299)
Supplement: Supplementary file 1 [file Table_1.DOCX]

**Supporting Information**

**Length-dependent Structural Transformations of Huntingtin PolyQ Domain Upon Binding to 2D-nanomaterials**

Mei Feng^1,*^, David R. Bell^2^, Zhenhua Wang^1^, Wei Zhang^3^

1. Institute of Quantitative Biology, Department of Physics, Zhejiang University, Hangzhou, 310027, China

2. Computational Biological Center, IBM Thomas J. Watson Research Center, Yorktown Heights, NY 10598, USA

3. School of Materials and Physics, China University of Mining and Technology, Xuzhou 221116, China

^*^Correspondence and requests for materials should be addressed to M.F (Email:fengmei@zju.edu.cn )


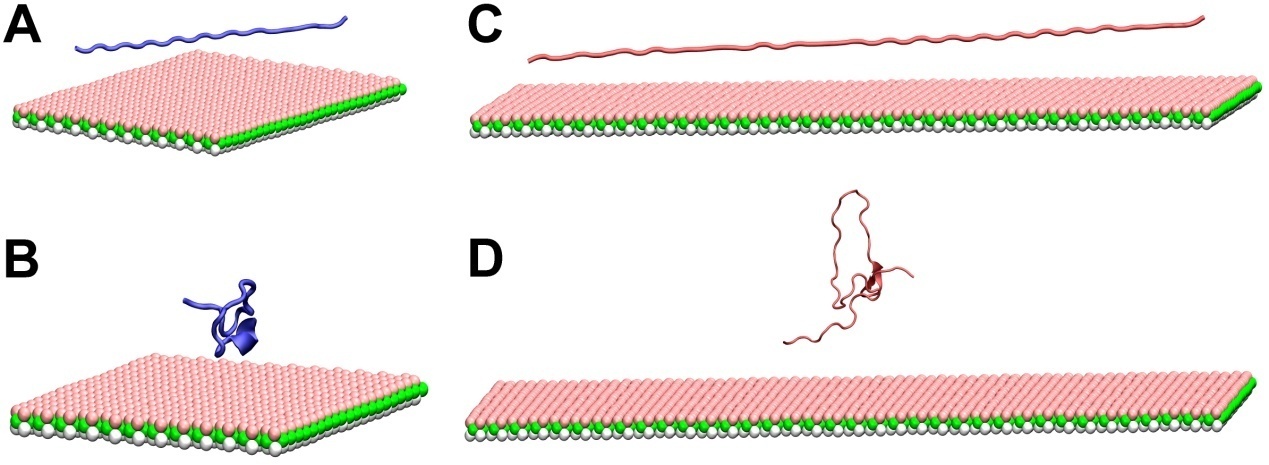


Figure S1. MD simulation systems of a fully extended (A) and collapsed (B) Q22 near the water–MoS2 interface, fully extended (C) and collapsed (D) Q46 near the water–MoS2 interface. Atoms in MoS_2_ and polyQ are shown as van der Waals Spheres and cartoon, respectively. Water molecules are not shown. The Q22 is colored in blue; and the Q46 is colored in red.


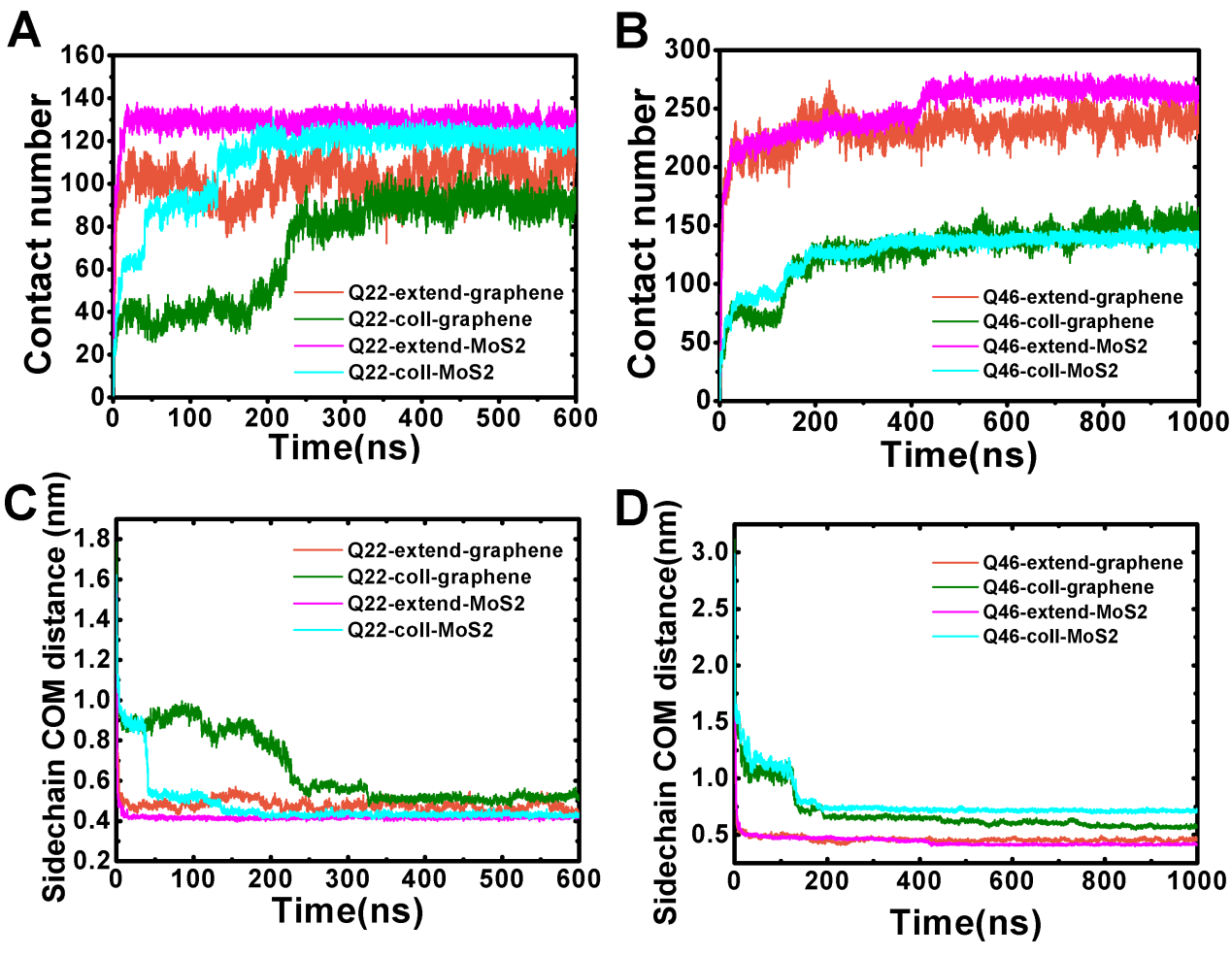


Figure S2. Atom contact number between polyQ and nanosheets for Q22 (A) and Q46 (B) as a function of simulation time. Average COM distances between polyQ sidechains and nanosheets for Q22 (C) and Q46 (D) as a function of simulation time. For MoS2, distances and contacts were computed to the S layer in the MoS2 sheet.


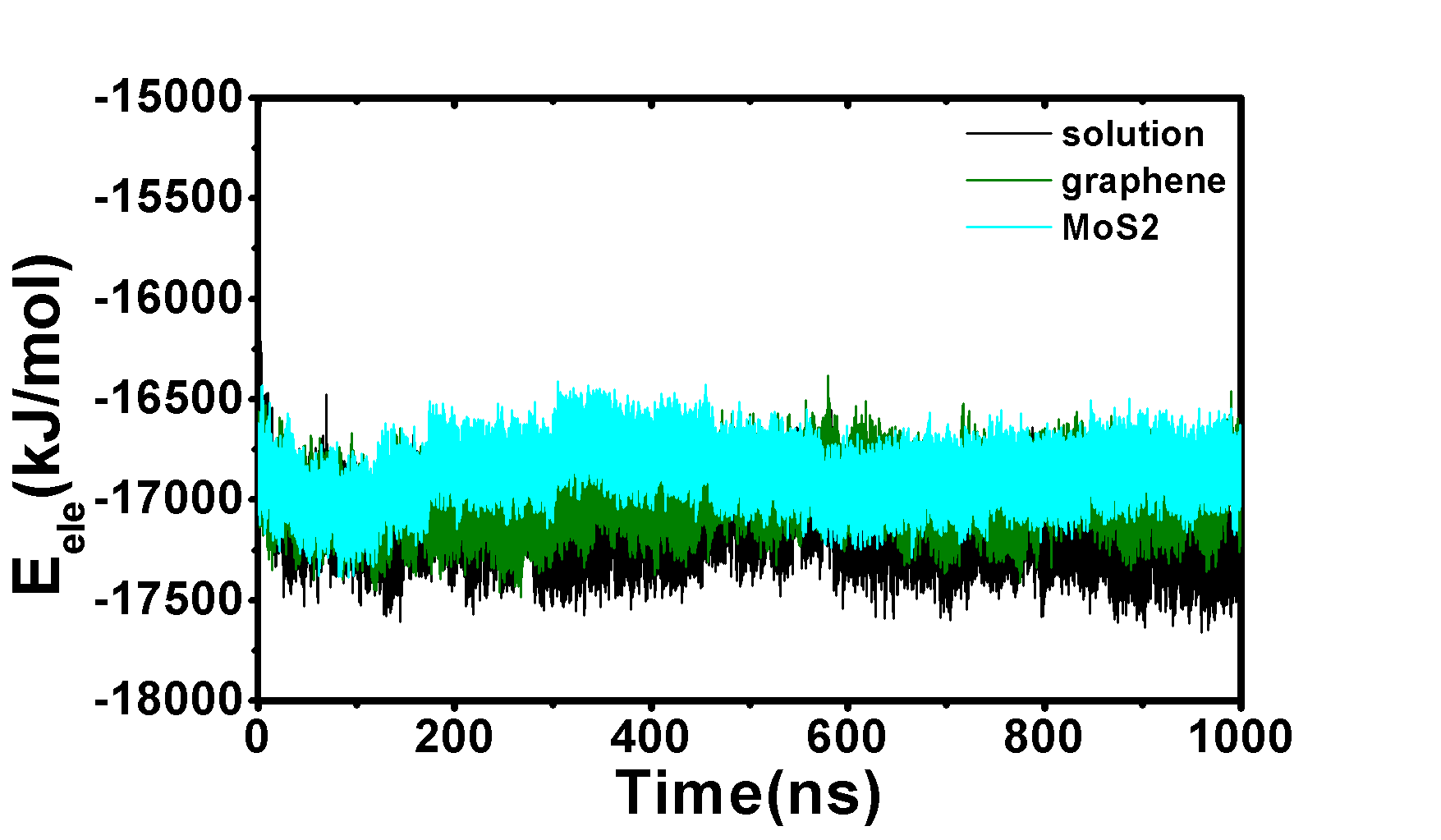


Figure S3. The Q46 self electrostatic interaction energy analyses of initially collapsed Q46 alone in water (black), on graphene (green), and MoS2 (blue) nanosheets.


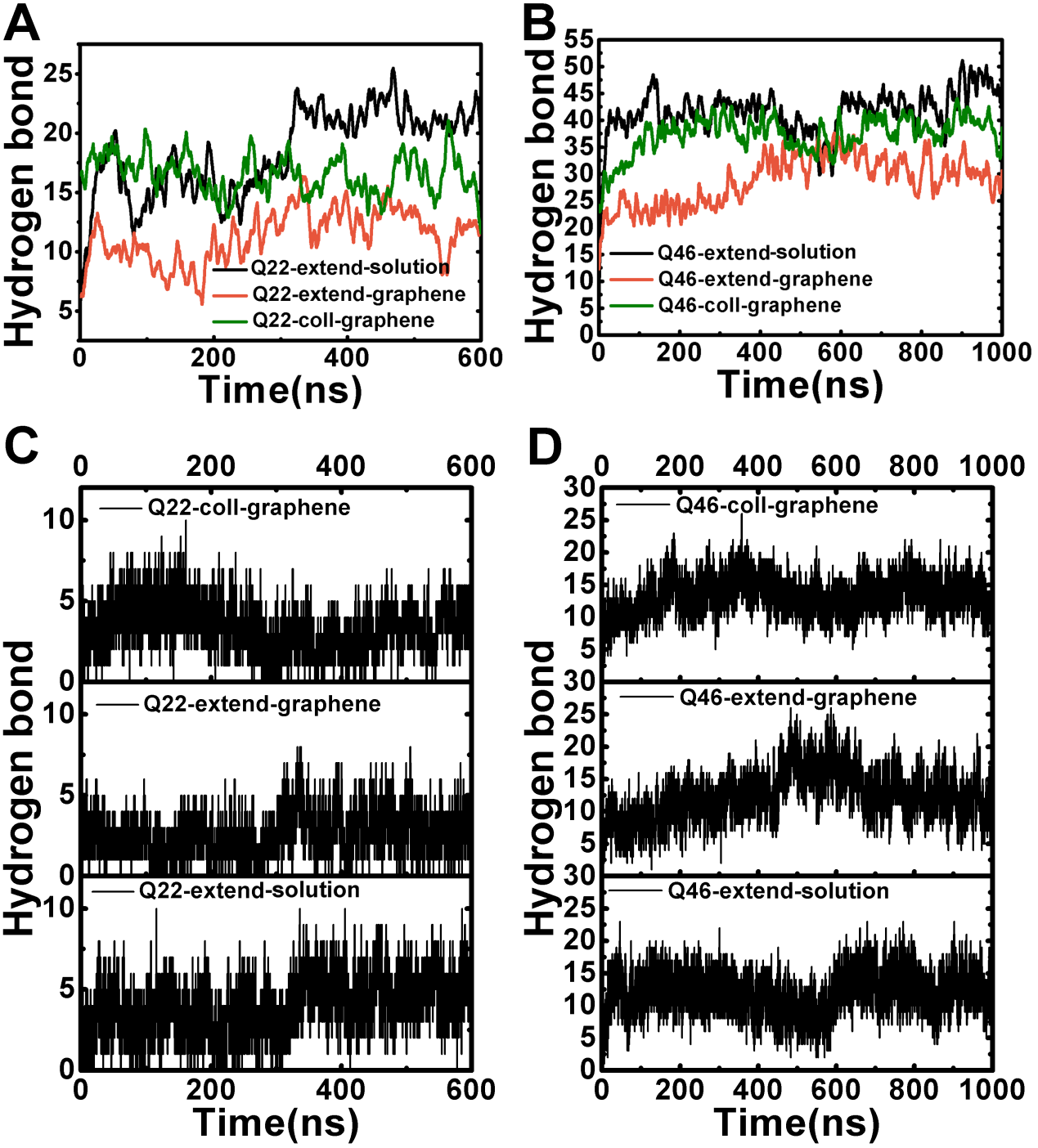


Figure S4. The number of hydrogen bonds inside Q22 (A) and Q46 (B) , and the number of side chain-side chain hydrogen bonds for Q22 (C) and Q46 (D) over the course of the graphene simulations.


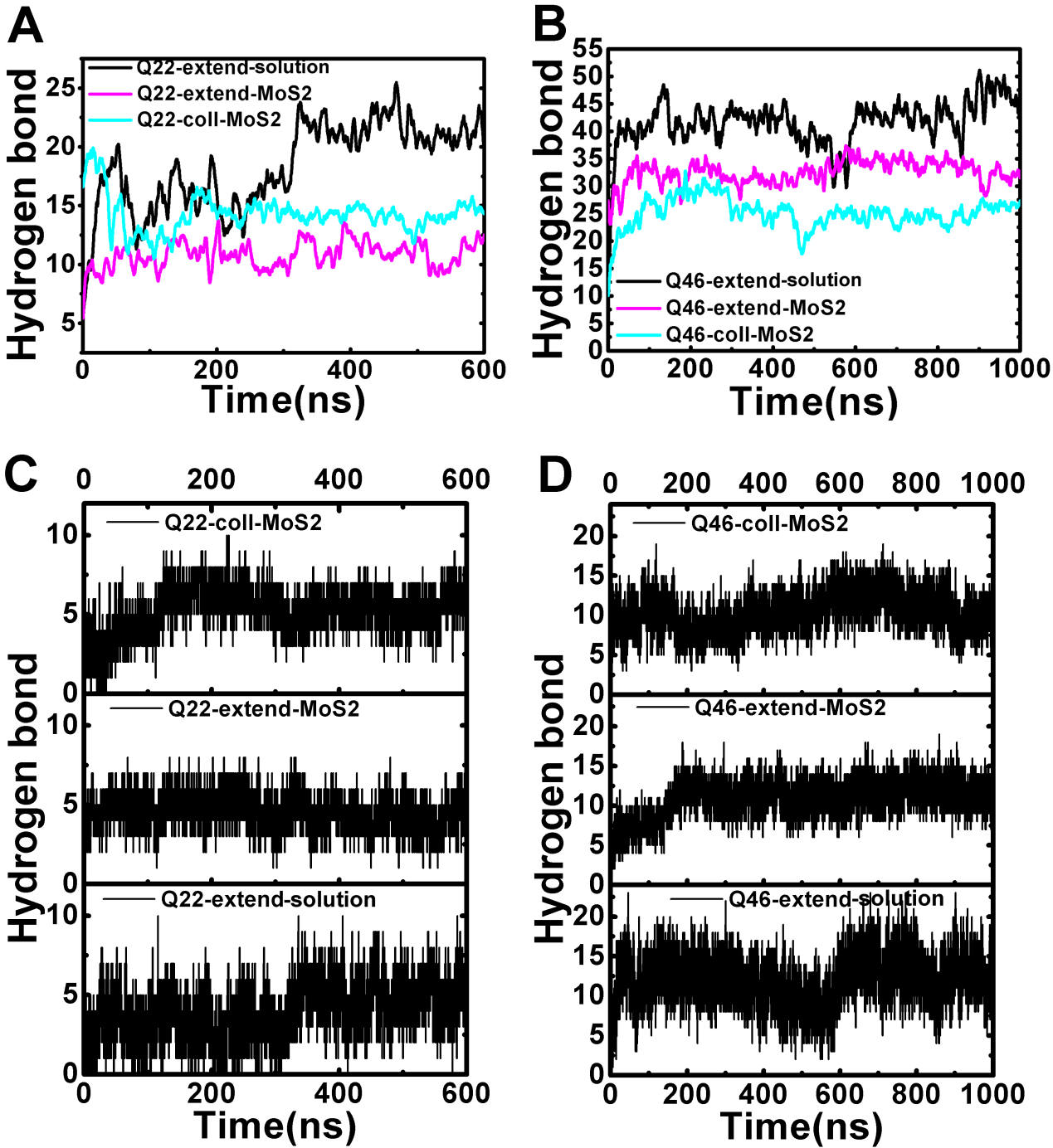


Figure S5. The number of hydrogen bonds inside Q22 (A) and Q46 (B) , and the number of sidechain-sidechain hydrogen bonds for Q22 (C) and Q46 (D) over the course of the MoS2 simulations.
